# Stereotaxic Diffusion Tensor Imaging White Matter Atlas for the *in vivo* Domestic Feline Brain

**DOI:** 10.3389/fnana.2020.00001

**Published:** 2020-02-11

**Authors:** Philippa J. Johnson, Raluca Pascalau, Wen-Ming Luh, Ashish Raj, Sofia Cerda-Gonzalez, Erica F. Barry

**Affiliations:** ^1^Department of Clinical Sciences, College of Veterinary Medicine, Cornell University, Ithaca, NY, United States; ^2^Faculty of Medicine, “Iuliu Hatieganu” University of Medicine and Pharmacy, Cluj-Napoca, Romania; ^3^National Institute on Aging, National Institutes of Health, Baltimore, MD, United States; ^4^Department of Radiology and Biomedical Imaging, University of California, San Francisco, San Francisco, CA, United States; ^5^Medvet Chicago, Northbrook, IL, United States

**Keywords:** cat, FA, MD, RD, AD, tractography, deterministic, connectome

## Abstract

The cat brain is a useful model for neuroscientific research and with the increasing use of advanced neuroimaging techniques there is a need for an open-source stereotaxic white matter brain atlas to accompany the cortical gray matter atlas, currently available. A stereotaxic white matter atlas would facilitate anatomic registration and segmentation of the white matter to aid in lesion localization or standardized regional analysis of specific regions of the white matter. In this article, we document the creation of a stereotaxic feline white matter atlas from diffusion tensor imaging (DTI) data obtained from a population of eight mesaticephalic felines. Deterministic tractography reconstructions were performed to create tract priors for the major white matter projections of Corpus callosum (CC), fornix, cingulum, uncinate, Corona Radiata (CR), Corticospinal tract (CST), inferior longitudinal fasciculus (ILF), Superior Longitudinal Fasciculus (SLF), and the cerebellar tracts. T1-weighted, fractional anisotropy (FA), mean diffusivity (MD), radial diffusivity (RD) and axial diffusivity (AD) population maps were generated. The volume, mean tract length and mean FA, MD, AD and RD values for each tract prior were documented. A structural connectome was then created using previously published cortical priors and the connectivity metrics for all cortical regions documented. The provided white matter atlas, diffusivity maps, tract priors and connectome will be a valuable resource for anatomical, pathological and translational neuroimaging research in the feline model. Multi-atlas population maps and segmentation priors are available at Cornell’s digital repository: https://ecommons.cornell.edu/handle/1813/58775.2.

## Introduction

The domestic cat (*Felis catus*) is a useful animal model for neuroanatomical, electrophysiological and neuropathological research (Preuss, 2000; Haller, 2013; Chambers et al., 2015; Kumar et al., 2016). Rapid advancements in functional and structural magnetic resonance imaging (MRI) techniques have enhanced our ability to evaluate the brain and there are many potential applications for these techniques in neuroscientific research where the domestic cat is a commonly used model.

Stereotaxic brain atlases play an important role in advanced neuroimaging research, being widely used for registration, identification and reporting cortical locations in a common coordinate system (Mori et al., 2008). For the cat, a three-dimensional (3D) cortical atlas and tissue probability maps of the brain have recently become available (Stolzberg et al., 2017). This atlas was created by the linear registration of T1-weighted structural MRI data and is a useful tool for normalization and segmentation of feline brain data. However, due to the limited ability for T1-weighted structural imaging data to evaluate the white matter, this atlas provides little information on white matter pathways and sub-regions and in the feline brain (Toga et al., 2006).

Diffusion tensor imaging (DTI) is a technique that is able to detect the characteristics of water diffusion within brain tissue (Alexander et al., 2007). White matter is composed of linear fascicle bundles, within which, water diffuses in a highly anisotropic fashion. DTI has been developed specifically to measure the random Brownian motion of water molecules in the body (Basser and Özarslan, 2014). This method is able to document the diffusivity characteristics of white matter tissue as well as inform on other neurological phenomena such as edema, infarction, and stroke by measuring the displacement of water molecules over time (Moseley et al., 1990; Jones, 2014). By using DTI, it is possible to infer anatomical characteristics of the underlying tissues per voxel (Smith et al., 2004). DTI is able to detect the orientation of this diffusion and by doing so, map the structure of white matter fibers within the brain in a method called tractography (Basser et al., 1994). There are two major forms of tractography; deterministic and probabilistic (Jones, 2008). Deterministic tractography assumes that there is a single dominant diffusion direction and links these dominant directions to create a tract. This technique is commonly used in neuro-navigation systems due to its simplicity and rapid results however it does have limitations in resolving curving, crossing or kissing tracts. Probabilistic tractography uses a complex algorithm to trace several thousand possible pathways from a starting seed region. This produces a highly sensitive probability distribution of connections that can be thresholded to include only the most likely connections (Jones, 2008). Both these forms of tractography have been used to create functional white matter brain maps that are routinely used, in the human, rhesus macaque, rat, mouse, dog and ferret research (Mori et al., 2008; Jiang and Johnson, 2011; Rumple et al., 2013; Zakszewski et al., 2014; Calabrese et al., 2015; Robinson et al., 2016; Hutchinson et al., 2017). Tractography techniques have been used to document the anatomy of feline white matter tracts (Jacqmot et al., 2017) and evaluate the development and regional variation of white matter in the juvenile feline brain (Takahashi et al., 2009; Dai et al., 2016). These articles have provided information on the location and diffusivity characteristics of several pathways; however, these previous articles do not compare *in vivo* tract reconstructions with *ex vivo* gross anatomic dissection. In addition, while previous articles provide a description of several tracks they do not provide open source diffusivity maps or tract-based priors of their atlases.

To enable automated segmentation of the white matter in the cat a white matter atlas and anatomic priors are required. In this study, we develop and make available a white matter brain atlas and structural connectome using DTI MRI data obtained *in vivo* at 3-tesla field strength from eight neurologically normal felines. We performed the tract reconstructions using deterministic tractography and the resulting tractography reconstructions are then compared directly to previously reported white matter gross anatomic dissections based on the Klingler method (Pascalau et al., 2016, 2018). We provide diffusivity maps and create a structural connectome to assess whole-brain connectivity. This comprehensive atlas will provide a valuable resource for scientists doing anatomical, pathological and translational neuroimaging research in the feline model.

## Materials and Methods

### Animals and Anesthesia

This study was approved by Cornell University’s Institutional Animal Care and Use Committee. Eight neurologically normal, mature (age at scanning mean = 1.52 years, standard deviation = 1.02 years), mixed-sex (four male and four female), domestic short-haired felines were recruited from research populations. After pre-anesthetic evaluations, each subject underwent intra-venous catheterization, pre-medication with dexmedetomidine (10 mg/kg) and was induced to general anesthesia with Propofol (10–20 mg/kg dosed to effect). All subjects were intubated and maintained under anesthetic with inhalant isoflurane/oxygen and supportive intravenous lactated ringer’s solution fluids. All protocols were approved by a board-certified veterinary anesthesiologist (American College of Veterinary Anesthesia).

### Image Acquisition

Imaging was performed in a General Electric Discovery MR750 3-Tesla unit (60 cm bore diameter), operating at 50 mT/m amplitude and 200 T/m/s slew-rate. Subjects were placed in dorsal recumbency with their head centered in a 16-channel small flex radio-frequency coil (NeoCoil, Pewaukee, WI, USA). The diffusion-tensor images were acquired using a single-shot echoplanar imaging DTI sequence that was acquired in the axial plane (TR = 7,000 ms, TE = 91.7 ms, flip angle = 90°, resolution = 1.5 mm^3^, slice thickness 1.5 mm, matrix size = 96 × 96 × 48, slice number 48, acquisition time 7 min and 42 s) with 60 gradient directions and a single unweighted diffusion image. The *b*-value was set to 800 s/mm^2^ within normal ranges for feline diffusion imaging. A high-resolution T1-weighted 3D inversion-recovery fast spoiled gradient echo sequence (Bravo) was performed in each subject with the following parameters; Isotropic voxels 0.5 mm^3^, TE = 3.6 ms, TR = 8.4 ms, TI = 450 ms and a flip angle of 12°.

### T1-Weighted Image Preprocessing and Population Template Creation

A study-specific T1-weighted template was created to allow for neuroanatomically informed tractography seed mask placement and anatomic referencing. T1-weighted images were corrected for low-frequency intensity inhomogeneity (Zhang et al., 2001). Initially, the Brain Extraction Toolbox (Smith, 2002) from the FSL toolbox (Smith et al., 2004; Woolrich et al., 2008; Jenkinson et al., 2012) was used to extract brain tissue from the skull, however since this algorithm is not optimized for canine brain extraction the brain mask was incomplete therefore manual removal of non-brain tissues and inclusion of brain issue was applied prior to the images being affine (linearly) registered using FMRIB’s Linear Image Registration Tool (FLIRT; Jenkinson and Smith, 2001; Jenkinson et al., 2002) and spatially normalized which involved registering each individual subject to a population template details of the methodology is described in the following article (Friston et al., 1995). The origin of the images was set to the rostral commissure, then the data were reoriented to a standard FMRI Software Library (FSL) orientation for consistency (Jenkinson et al., 2012). After the creation of the T1 template, it was linearly registered to the diffusion population template using ITK-SNAP (Yushkevich et al., 2006) to provide a high-resolution anatomical reference.

### DWI Image Preprocessing

**Figure 1:**
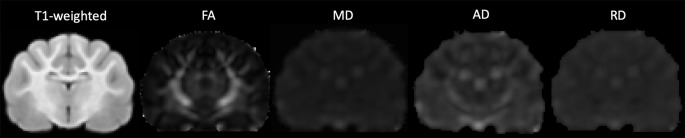
The transverse image at the mid-brain level of the T1-weighted population template and fractional anisotropy (FA), mean diffusivity (MD), axial diffusivity (AD) and radial diffusivity (RD) diffusivity maps.

DWI images were corrected for noise using principal component analysis (PCA) to estimate a noise map as described in detail in the following article (Veraart et al., 2016a,b). Corrections for phase distortion (Andersson et al., 2003; Smith et al., 2004), eddy current distortion and motion (Andersson and Sotiropoulos, 2016) were also implemented using the FSL^1^ and MRTrix^2^ software packages. Initially, a brain mask was estimated from the subject’s diffusion data using dwi2mask command as part of the MRTrix toolbox (Tournier et al., 2012) however this masking was sub-optimal for feline subjects. Therefore, manual brain tissue segmentation was additionally applied by an experienced analyst (EFB) to remove non-brain tissue. Brain masks were then viewed and validated by an independent rater (PJJ). Diffusion tensor measures including fractional anisotropy (FA), mean diffusivity (MD), axial diffusivity (AD) and radial diffusivity (RD) were calculated using the FSL FMRIB’s Diffusion Toolbox (Behrens et al., 2007). Diffusion tensor maps for each diffusivity parameter were obtained for each participant and visually inspected for quality assurance (Figure 1).

### DWI Population Template Creation

To create the population DWI template three types of registration were tested to identify the method created the optimal template. Both FLIRT (FMRIB’s Linear Image Registration Tool) and non-linear methods (ANTs) were applied to the data. After preprocessing, each subject’s data were registered to a common space and anatomically aligned using restricted linear registration FLIRT with 6° of freedom. With the exact alignment of each subject’s brain, using the T1 weighted high-resolution data, non-linear registration and template creation using Advanced Normalization Tools (ANTs), was found to be the best method of population template creation (Avants et al., 2011). The resulting DWI template was then used for the deterministic tractography. A population template for fiber orientation distribution (FOD) images was processed and created with MRtrix for the creation of a structural connectome (Tournier et al., 2007).

### White Matter Tractography

White matter tract reconstructions were performed using the FACT algorithm (Mori et al., 1999) with a step length of 0.5 mm and angle threshold of 35° in TrackVis (Wang et al., 2007) within a binary brain mask. The ROI seed placement adapted from that described by Catani and Thiebaut de Schotten (2008) and feline tractography descriptions (Dai et al., 2016; Jacqmot et al., 2017). Seed regions were delineated on the FA maps and once tracts were generated obvious spurious tracts were removed. All tracts were reviewed by a white matter anatomy expert (PR) and compared to previously published gross anatomic dissections (Pascalau et al., 2016). A description of each tract reconstruction follows:

### Associative Tracts

#### Superior Longitudinal Fasciculus (SLF)

The superior longitudinal fasciculus (SLF) is an associative tract composed of both short and long fibers that form connections within the perisylvian cortex. It occupies a dorsal and lateral position in the brain. It was reconstructed using a single arcuate ROI placed over the mid and dorsal arcuate gyri, lateral to the Corona Radiata (CR) on the coronal plane image.

#### Inferior Longitudinal Fasciculus (ILF)

The inferior longitudinal fasciculus (ILF) is a caudoventrally located associative tract that connects the occipital (extrastriate cortex) and temporal (temporal pole, middle and inferior gyri) lobes. This tract was reconstructed using two ROIs; an occipital ROI was placed over the occipital lobe on the coronal plane and a temporal ROI that spanned the mid-temporal lobe ventral and lateral to the external capsule on the coronal plane.

#### Uncinate Fasciculus (UF)

This is a small rostroventrally located associative tract that connects the rostral temporal lobe with the orbitofrontal cortex. This tract was reconstructed using a two ROI approach with one ROI placed over the rostral temporal lobe on the coronal plane and the other placed over the frontal white matter on the transverse plane.

#### Cingulum

A medially located associative tract with variably sized fibers connecting the frontal, parietal, occipital and temporal lobes within the cingulate gyrus. It was reconstructed using a single ROI placed along the length of the linear cingulum, immediately dorsal to the body of Corpus callosum (CC), to ensure both long and short fibers are incorporated.

### Commissural Tract

#### Corpus Callosum (CC)

The CC is an extensive tract that connects homologous cortical areas on the right and left cerebral hemispheres. It was reconstructed using a single ROI method; a CC ROI was placed on the sagittal plane covering the body of CC at the mid-sagittal section.

### Projection Tracts

#### Fornix

This central projection tract connects the mammillary bodies and hippocampus. It was reconstructed using a ROIs placed over the body and crura of the fornix, on the coronal plane, ventral to the CC.

#### Corona Radiata (CR)

This large projection system has fibers that ascend from the thalamus to the cerebral cortex, occupying the most medial position in the internal capsule. It was reconstructed by placing an ROI on the coronal plane over the internal capsule and applying an exclusion mask to remove fibers that extended the caudal to the thalamus.

#### Corticospinal Tract (CST)

This white matter pathway extends between the frontoparietal cortex and the spinal cord. It was reconstructed using a two ROI approach, with one placed over the internal capsule and the other further caudally within the ventral brainstem.

### Cerebellar Tracts

#### Cerebellar Thalamocortical Tract (CTCT)

This is the efferent cerebellar tract and extends from the cerebellum, through the superior cerebellar peduncle, red nucleus and thalamus to terminate within the cerebral cortex (Habas and Manto, 2018). This tract was reconstructed by placing a rounded ROI over the superior cerebellar peduncle, at the level of the midbrain, on the coronal plane and a second ROI at the level of the internal capsule (Catani and Thiebaut de Schotten, 2008).

#### Dorsal Spinal-Cerebellar Tract (DSCT)

This is one of the afferent cerebellar tracts. It extends from the spinal cord through the inferior cerebellar peduncle. It was reconstructed using rounded ROIs placed on the coronal plane over the inferior cerebellar peduncles, lateral to the medulla oblongata and a second ROI placed over the caudal brain stem (Catani and Thiebaut de Schotten, 2008).

#### Ventral Spinal-Cerebellar Tract (VSCT)

This cerebellar tract extends from the ventral spinal cord into the superior cerebellar peduncle and into the ipsilateral cerebellum. It was reconstructed using a two ROI technique, one placed on the coronal plane over the superior cerebellar peduncle and the other placed on the axial plane over the spinal cord.

#### Pontocerebellar Tract (PCT)

This cerebellar tract contains efferent and afferent fibers and extends to the pons and further rostrally along the ventrolateral aspect of the mesencephalon (Habas and Manto, 2018). It was reconstructed using rounded ROIs placed on the coronal plane over the middle cerebellar peduncles (Catani and Thiebaut de Schotten, 2008).

### Anatomic Prior Diffusivity and Volume

For each white matter tract fiber length (mean and standard deviation obtained from TrackVis software) and tract volumes were documented. In order to document diffusivity values, a white matter skeleton was created from the FA maps by thresholding to remove voxels that extended into gray matter (FA <0.15). This skeleton was applied to the tract masks to constrain these masks to the white matter only. The final tract masks were then applied to the diffusion tensor maps of FA, MD, RD, and AD to calculate the mean and standard deviation of these tensor measures within the tracts using FSL tools (Smith et al., 2004).

### Connectome

Diffusion MRI can allow for the construction of a brain connectome on a macroscopic scale to explore the connectivity between regions and inter-regional pathways. A structural brain connectome is a collection of white matter pathways (edges) that connect a set number of regions of interests (nodes; Rubinov and Sporns, 2010). These white matter pathways are based on selecting a number of streamlines connecting two regions (Hagmann et al., 2010). The number of streamlines selected and their trajectory is determined by the choice of tractography algorithm (Tournier et al., 2011). In order to generate a structural connectome the following procedures were implemented. The individual subjects’ diffusion and structural data were processed in accordance with methods for the template reconstruction (see above). After preprocessing, global intensity normalization was applied to the diffusion data in order to reduce intensity bias across subjects. The subjects’ data were then up-sampled to a resolution of 0.5 mm^3^. The global intensity normalization process produced an FA and white matter mask template (thresholded at 0.2). From the normalized diffusion data across subjects a FOD template was created by estimating and averaging the response function of each subject than using constrained spherical deconvolution to create a group FOD template (Tournier et al., 2004). The FOD brain mask was created by registering each subjects’ processed diffusion data to the FOD template, saving the registration matrices, then applying these matrices to the subjects’ diffusion brain mask to create an averaged brain mask (Raffelt et al., 2011, 2012). This brain mask was visually inspected for any anatomical inaccuracies and was manually corrected to include all brain regions. A twenty million tract whole-brain tractogram was then created from the FOD template using the iFOD2 (Second-order Integration over Fiber Orientation Distributions; Tournier et al., 2010, 2012) which included a maximum angle of 35°, maximum tract length of 250, a minimum tract length of 10, and power of 1. The is weights were created using MRTrix3 spherical-deconvolution informed filtering of tractograms version 2 (SIFT2) by determining appropriate cross-sectional area multiplier for each streamline (Smith et al., 2015). This step allows for a more biologically accurate fiber connectivity measure and is further detailed in Smith et al. (2015). The CATLAS (Stolzberg et al., 2017) was then registered to the FOD template space using ITK-SNAP manual linear registration (Yushkevich et al., 2006). The transformed CATLAS regions were then used as nodes and labels for connectome generation. The structural connectivity matrix was generated using the 2 million track tractogram with SIFT2 track weights and CATLAS regions as nodes. This connectome included 151 regions as per the original CATLAS using the default streamline-parcel assignment mechanisms (Smith et al., 2015). The resulting connection strengths were divided by the sum of the voxels for both the seed and target regions to account for differences in volume between regions (Owen et al., 2013). The whole-brain weighted connectivity matrix was used to calculate connectivity metrics such as degree (the number of in and out links of a node), betweenness centralit (a measure of hub centrality based on the number of shortest paths through a node), strength (the sum of the weights of links connected to the node), and clustering coefficient (the fraction of triangles around each node relating the fraction of nodes neighbors who are also neighbors) generated and visualized using the Brain Connectivity Toolbox (BCT^3^) in MATLAB (Rubinov and Sporns, 2010) and MRtrix (Tournier et al., 2012).

## Results

### White Matter Atlas

Population maps for T1-weighted, FA, MD, AD and RD data are provided (Figure 1). Individual tract priors for the association, projection, commissural and cerebellar tracts were generated. The volumes, mean fiber lengths and MD parameters for each tract are provided in Table 1. The atlas is made available as an online resource at the following web address: https://ecommons.cornell.edu/handle/1813/58775.2.

**Table 1:** Volume (mm^3^), fiber length (mean and standard deviation (st dev), fractional anisotropy (FA; mean and st dev), mean diffusivity (MD; mean and st dev), axial diffusivity (AD; mean and st dev) and radial diffusivity (RD; mean and st dev) of each reconstructed tract.

	Volume (mm^3^)	Fiber Length (mm)		FA		MD		AD		RD	
		Mean	St Dev	Mean	St Dev	Mean	St Dev	Mean	St Dev	Mean	St Dev
Left SLF	1,165.6	11.8	7.2	0.208	0.053	0.000061	0.00001	0.000074	0.000012	0.000055	0.000009
Right SLF	905.4	11.6	7.2	0.203	0.047	0.000061	0.000008	0.000074	0.000011	0.000055	0.000007
Left ILF	172.0	42.4	21.1	0.315	0.090	0.000062	0.000006	0.000084	0.000009	0.000051	0.000007
Right ILF	175.0	25.4	4.1	0.220	0.060	0.000062	0.000008	0.000077	0.000011	0.000055	0.000008
Left Uncinate	271.5	21.1	5.3	0.203	0.050	0.000072	0.000049	0.000088	0.00006	0.000064	0.000043
Right Uncinate	259.1	17.7	3.1	0.207	0.049	0.00005	0.000008	0.000061	0.00001	0.000044	0.000008
Left Cingulum	469.7	15.7	9.2	0.187	0.034	0.000058	0.000004	0.00007	0.000005	0.000052	0.000004
Right Cingulum	559.7	16.5	11.6	0.201	0.045	0.00006	0.000008	0.000074	0.00001	0.000054	0.000007
CC	2,568.1	30.0	15.1	0.222	0.060	0.000061	0.000007	0.000075	0.000009	0.000053	0.000007
Fornix	574.4	40.4	3.0	0.201	0.043	0.000064	0.000008	0.000079	0.00001	0.000057	0.000008
Left CR	1,110.0	23.8	7.7	0.286	0.091	0.00006	0.000011	0.000079	0.000015	0.000051	0.00001
Right CR	810.2	25.3	7.9	0.278	0.095	0.000059	0.000009	0.000077	0.000012	0.00005	0.000009
Left CST	844.8	47.7	7.7	0.315	0.114	0.000059	0.000019	0.000081	0.000026	0.000048	0.000017
Right CST	1,052.5	49.6	6.2	0.310	0.118	0.000059	0.000013	0.00008	0.000019	0.000048	0.000012
Left DSCT	187.5	13.4	5.1	0.221	0.058	0.000048	0.000014	0.000059	0.000017	0.000042	0.000012
Right DSCT	81.5	11.8	2.7	0.249	0.069	0.000052	0.000013	0.000066	0.000017	0.000045	0.000012
Left CTCT	386.7	47.8	5.5	0.299	0.117	0.000061	0.000011	0.000081	0.000016	0.00005	0.000011
Right CTCT	389.1	47.3	2.8	0.309	0.133	0.000059	0.00001	0.000079	0.000014	0.000048	0.000011
Left PCT	556.1	29.6	4.2	0.253	0.084	0.000068	0.000021	0.000087	0.000025	0.000059	0.000019
Right PCT	429.0	32.8	7.9	0.225	0.058	0.000065	0.00002	0.000082	0.000027	0.000057	0.000019

*SLF, superficial longitudinal fasciculus; ILF, inferior longitudinal fasciculus; CC, corpus callosum; CR, corona radiata; CST, corticospinal tract; DSCT, dorsal spinal-cerebellar tract; CTCT, cerebellar thalamocortical tract; PCT, pontocerebellar tract*.

### Associative Tracts

#### Superior Longitudinal Fasciculus (SLF)

**Figure 2:**
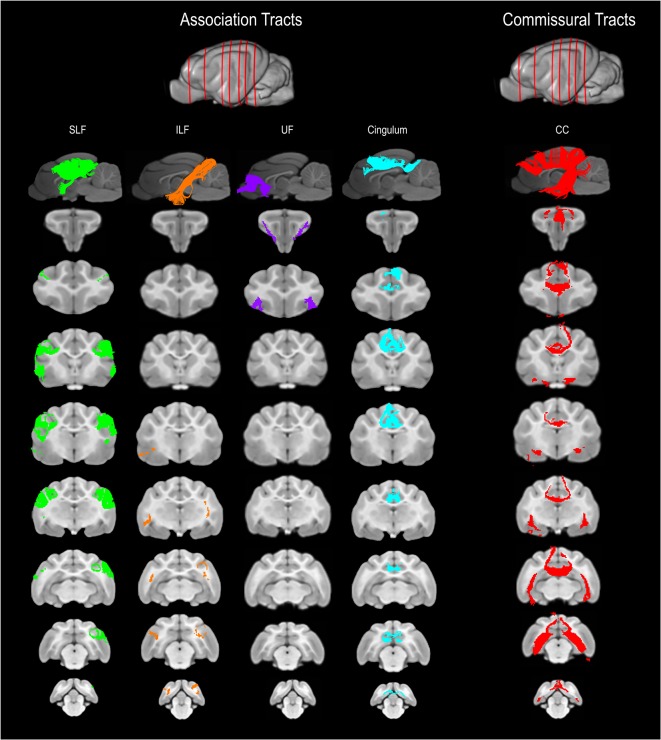
Three-dimensional (3D) and 2-dimensional (2D) visual documentation of the association and commissural tract reconstructions overlaid on T1-weighted structural images in sagittal and transverse planes with slice reference images. SLF, superficial longitudinal fasciculus; ILF, inferior longitudinal fasciculus; UF, uncinate fasciculus; Cingulum and CC, corpus callosum.

The SLF had an appropriate lateral position and exhibited fronto-parietotemporal connections (Figure 2). It ran in a frontoparietal direction, encircling the external capsule, consistent with that described on gross anatomic dissection (Pascalau et al., 2016). When compared to gross anatomic dissections the size, location, course and connectivity of the SLF were very similar (Figures 4A–C). The right SLF was slightly larger than the left side (1,510.1 mm^3^ and 1,165.6 mm^3^ respectively), however, the mean tract length was longer on the left side than the right (11.8 mm and 11.4 mm respectively; Table 1).

**Figure 3:**
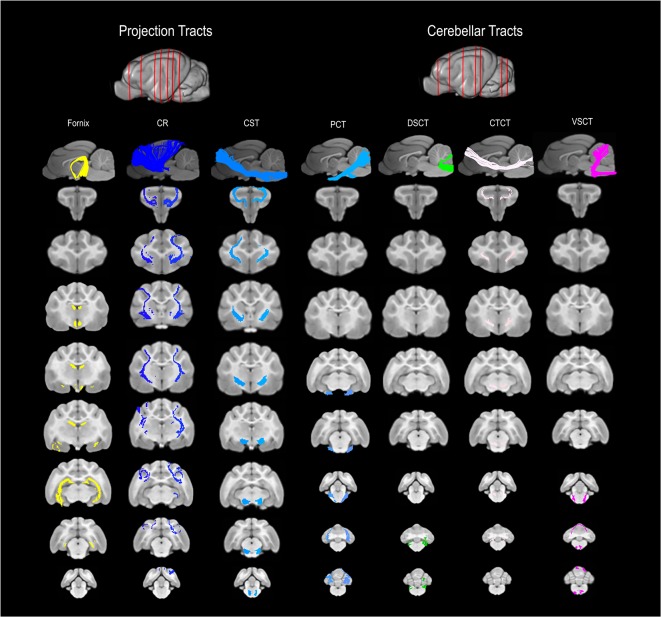
3D and 2D visual documentation of the projection and cerebellar tract reconstructions overlaid on T1-weighted structural images in sagittal and transverse planes with slice reference images. Project Tracts included Fornix; CR, corona radiate; CST, corticospinal tract. Cerebellar Tracts included PCT, pontocerebellar tract; DSCT, dorsal spinal-cerebellar tract; VSCT, ventral spinal-cerebellar tract and CTCT; cerebellar thalamocortical tract.

**Figure 4:**
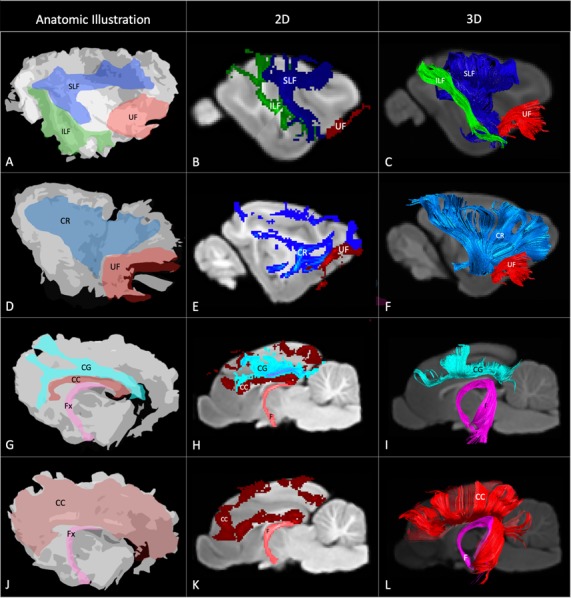
Demonstrates anatomic illustrations (left column) and the 2D and 3D images of the associated white matter tract reconstructions overlaid on sagittal plane T1-weighted images. **(A)** Gross anatomic dissection illustration of the SLF, superficial longitudinal fasciculus; ILF, inferior longitudinal fasciculus; and UF, uncinate fasciculus. **(B)** 2D images of the SLF, ILF and UF overlain on a T1-weighted parasagittal slice. **(C)** 3D images of the SLF, ILF and UF overlain on a T1-weighted para-sagittal slice. **(D)** Gross anatomic dissection illustration of the CR, corona radiata and UF. **(E)** 2D images of the CR and UF overlain on a T1-weighted parasagittal slice. **(F)** 3D images of the CR and UF overlain on a T1-weighted para-sagittal slice. **(G)** Gross anatomic dissection illustration of the CG, cingulum; CC, corpus callosum and f, fornix. **(H)** 2D images of the CG, CC and F overlain on a T1-weighted mid-sagittal slice. **(I)** 3D images of the CG and F overlain on a T1-weighted mid-sagittal slice. **(J)** Gross anatomic dissection illustration of the CC and F. **(K)** 2D images of the CC and F overlain on a T1-weighted mid-sagittal slice. **(L)** 3D images of the CC and F overlain on a T1-weighted mid-sagittal slice. Illustrations created from previously published gross anatomic white matter dissections with author consent (Pascalau et al., 2016).

#### Inferior Longitudinal Fasciculus (ILF)

The ILF extended between the occipital and temporal regions and was located caudal to the SLF (Figure 2). It contained long occipito-temporal fibers as well as shorter, more superficial fibers connecting adjacent areas. Its location was similar to that observed in gross anatomic dissections (Figures 4A–C). The reconstructed ILF tracts had the smallest volume of all included forebrain tracts and had similar volumes on both right and left sides (175.0 mm^3^ and 172.0 mm^3^ respectively; Table 1).

#### Uncinate Fasciculus (UF)

The uncinate fasciculus (UF) was observed to extend from the rostrolateral aspect of the temporal lobe to the frontal region (Figure 2). It had a similar location (lateral and ventral to the lenticular nucleus) and curvilinear shape to that observed in gross anatomic dissections (Figures 4A–F). These paired structures had similar volumes on the right and left sides (259.1 mm^3^ and 271.5 mm^3^ respectively; Table 1).

#### Cingulum

The cingulum tracked appropriately along the cingulate gyri and exhibited frontal connectivity consistent with that described in gross anatomic dissections and tractography (Dai et al., 2016; Pascalau et al., 2016; Figures 2, 4G–I). Mean tract length was relatively low [left 15.66 ± 9.2 and right (16.5 ± 11.6)], likely due to the short radiating fibers that extend dorsally from the cingulum (Table 1).

### Commissural Tract

#### Corpus Callosum (CC)

The reconstructed CC was the largest tract of the group (2,568.1 mm^3^) and demonstrated strong inter-hemispheric connection between occipital, frontal, parietal and temporal lobes (Figure 2, Table 1). It was well delineated from the adjacent cingulum and fornix (Figures 4J–L). Although the majority of the callosal fibers projected dorsally, in a concave fashion, there was a well-represented group of ventrally oriented fibers connecting the temporal lobes which correspond to the tapetum. This structure is similar to that observed in previous tractography descriptions (Dai et al., 2016).

### Projection Tracts

#### Fornix

The fornix formed distinct tracts extending around the thalamus, from the fimbria hippocampi to the subthalamic region (Figures 3, 4G–L). The reconstruction had a volume of 574.4 mm^3^ and was composed predominantly of longer fibers (mean fiber length 40.4 ± 3.0 mm; Table 1). The fibers are in close relation to the cerebral ventricles in multiple section planes. The structure of the fornix identified is consistent with that dissected anatomically and described with tractography (Dai et al., 2016; Jacqmot et al., 2017; Pascalau et al., 2018).

#### Corona Radiata (CR)

The CR formed a fan-shaped structure that extended within the internal capsule (Figure 3). It exhibited thalamocortical connectivity and extended to connect with the frontal, parietal and occipital regions (the later connections have a characteristic rostrocaudal direction and are known as the optic radiations (Pascalau et al., 2018; Figures 4D–F). The left CR was larger than the right side (1110.0 mm^3^ vs. 810.2 mm^3^ respectively; Table 1).

#### Corticospinal Tract (CST)

The corticospinal tract (CST) extended from the frontoparietal region and had an oblique path, passing through the cerebral peduncle (the ventral part of the midbrain) forming connectivity with the anterior horn of the spinal gray matter (Figure 3). These reconstructions had the longest fiber lengths of all dissected pathways (right; mean 49.6 ± 6.2 mm and left; mean 47.7 ± 7.7 mm; Table 1). The structure of the CST is similar to that observed in other tractography descriptions (Dai et al., 2016; Jacqmot et al., 2017).

### Cerebellar Tracts

#### Cerebellar Thalamocortical Tract (CTCT)

The CTCT exhibited frontoparietal connectivity and ran medially to the CST until the midbrain where it took a separate route to the cerebellum, passing through the superior cerebellar peduncle (Figure 3). Similar to the CSTs the CTCT reconstructions had high mean fiber lengths (left; 47.8 ± 5.5 mm and right; 47.3 ± 2.8 mm; Table 1).

#### Dorsal Spinal-Cerebellar Tract (DSCT)

The DSCT was the smallest of all cerebellar tract reconstructions (right 187.5 mm^3^ and left 81.5 mm^3^) due to truncation as the field of view ends (Figure 3, Table 1). It runs through the inferior cerebellar peduncles and occupies a dorsal position in the medulla oblongata and the spinal cord.

#### Ventral Spinal-Cerebellar Tract (VSCT)

The VSCT was identified extending out of the superior cerebellar peduncle before extending down the ventral aspect of the medulla oblongata and spinal cord (Figure 3).

#### Pontocerebellar Tract (PCT)

This was the largest cerebellar reconstruction and the left side was mildly larger than the right (left; 556.1 mm^3^, and right; 429.0 mm^3^; Figure 3, Table 1). It was the most laterally located cerebellar tract, emerging from the pons and spreading in the entire white matter domain of the cerebellar hemispheres.

### Connectome

**Figure 5:**
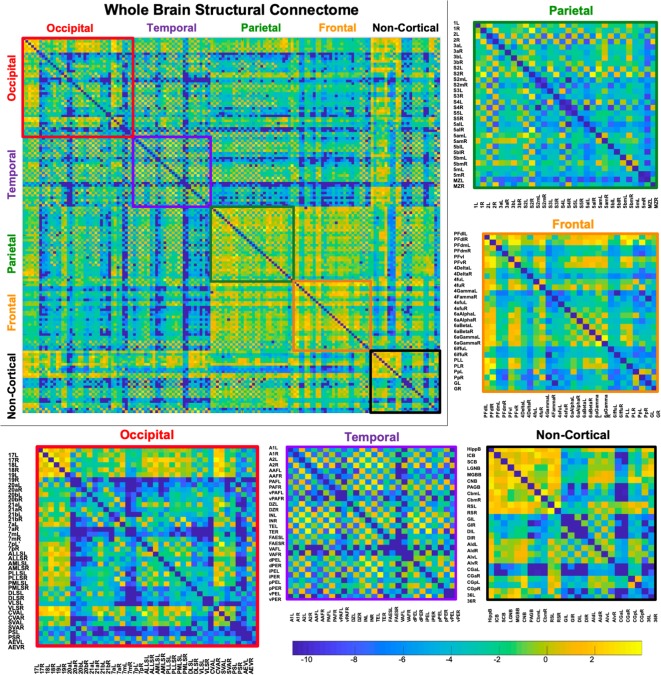
Demonstrates a weighted undirected structural connectome adjusted for voxel size on a log scale. The connectome has been color-coded according to lobe origin (red = occipital, purple = temporal, green = parietal, orange = frontal, and black = non-cortical). The individual lobar matrices have been enlarged and node names are listed on the x and y axes.

**Figure 6:**
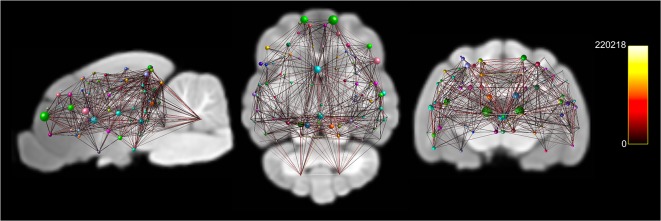
Documents node based weighted connectivity maps overlain on the population average T1-weighted structural image in sagittal, coronal and transverse planes. Node size corresponds to the connectivity strength (sum of weights of links connected to the node) of a particular node. Edge visualization was thresholded at 3,500 for visualization purposes. The connectome weight of each edge is colored by a heatmap with a minimum of 0 and maximum of 220,218 from the weighted connectome.

A whole-brain weighted undirected connectivity matrix that was adjusted for volume and node-based connectome maps are provided in Figures 5, 6. A reference table containing connectivity metrics for each cortical region is also provided in the Supplementary Table S1. This includes the region number, label and lobar parcellation and their corresponding betweenness centrality, degree of connectivity, clustering coefficient and strength.

## Discussion

In this article, we document the creation of a stereotaxic white matter atlas using DTI data from a group of healthy mesaticephalic cats. We provide downloadable FA, RD, AD, MD and T1-weighted brain maps and priors for projection, association, commissural and cerebellar associated white matter tracts and create a structural connectome with connectivity metrics.

Despite the cat being a widespread preclinical animal model in neuroscientific research, this species lacks the open-source stereotaxic MRI-based brain atlases available in other species (Mori et al., 2008; Jiang and Johnson, 2011; Rumple et al., 2013; Zakszewski et al., 2014; Robinson et al., 2016). The only open-source stereotaxic feline MRI atlas currently available is created with structural T1-weighted MRI data and focuses on cortical and deep gray-matter structures (Stolzberg et al., 2017). Our atlas provides a stereotaxic DWI-based atlas of the white matter in the feline brain. It complements the aforementioned feline T1 atlas and can be applied for anatomic registration and segmentation of the white matter to aid in lesion localization or standardized regional analysis of specific parts of the brain.

Up until now, a manual region of interest analysis has been exclusively used for the segmentation and evaluation of DWI data in feline studies (Zhao et al., 2006; Mizoguchi et al., 2017). This technique, however, has several drawbacks as it is time-consuming, prone to hypothesis-driven bias, and is poorly reproducible (Lilja et al., 2016; Peterson et al., 2016). Our atlas provides the tools required for registration and parcellation of the white matter into tract-based regions, for a more standardized, rapid and reproducible evaluation of the data.

Previous diffusion-based stereotaxic white-matter atlases have utilized multiple methods for segmentation. Within the white-matter, both location-based manual segmentations and connection-based tractography methods have been used to define anatomic regions and white-matter tracts (Mori et al., 2009; Hutchinson et al., 2017). Location-based atlases are created using manual segmentation of color FA maps with the guidance of histology-based atlases for anatomic reference to help delineate regions representing deep white matter locations (Mori et al., 2008, 2009). For our atlas we wanted to create segmentations that represented structural connections within the brain, applying insight from histology work and diffusion atlases from other species (Catani and Thiebaut de Schotten, 2008; Pascalau et al., 2016). Therefore we elected to use deterministic tractography to create a white matter atlas based on feline neuroanatomy and connectivity. Tractography provides a method that utilizes tissue diffusion characteristics to identify tract location and boundaries. This connection-based method of segmentation delineates tract margins, without the limitations of manual segmentation, and has been shown to accurately reflect the macroscopic anatomy of the major white matter pathways (Mori et al., 2009). To ensure anatomic accuracy of our reconstructions, where possible, we compared directly to gross anatomic dissection of the feline brain performed by the Klingler method, a tridimensional dissection technique which is complementary to tractography and has been used for validation of tractographic reconstructions mostly in human brain studies (Pascalau et al., 2016, 2018). This direct anatomic comparison was not found in other feline tractography papers (Dai et al., 2016; Jacqmot et al., 2017).

Our stereotaxic white matter atlas contains the major white matter pathways of the feline brain. Tract reconstructions were made using previously published tractography descriptions (Catani and Thiebaut de Schotten, 2008; Dai et al., 2016; Jacqmot et al., 2017). The resulting tractography reconstructions were reviewed for anatomic accuracy by a white matter anatomist (RP) and compared to gross anatomic white matter dissections. The inferior fronto-occipital fasciculus is not included in the atlas, as it was not identified even with liberal seed regions placed over the entire frontal and occipital regions. This fasciculus has been previously reconstructed in the feline using tractography (Jacqmot et al., 2017; Das and Takahashi, 2018). Although neuroanatomical connectivity analysis of the cat has shown both efferent and afferent connection between frontal and occipital regions (Scannell et al., 1995), the presence of a fronto-occipital fasciculus has not been identified on gross anatomic dissection (Pascalau et al., 2016). Histopathologic evaluation of the feline brain is required to confirm the presence or absence of this fasciculus in this species.

The diffusivity parameters we documented for each reconstructed tract prior are included in Table 1. Mean FA values have been documented for the developing feline brain and trended higher than our values (Dai et al., 2016). This may be due to the effect of subject age, differing acquisition technique, or be the consequence of taking averages from a population map rather than from individual subjects.

The structural connectome that was documented in this study provides an overview of the structural connectivity between cortical regions in the feline. Extensive neuroanatomic cortical-cortical connectivity analysis has been previously performed in the feline using electrophysiological methods (Scannell et al., 1995). These analyses identified that the sensory regions of the brain were connectionally isolated with stronger intra-lobar connectivity when compared to the connection between regions. This was thought to be either due to deletion of connections between lobes in the developing feline or be a consequence of experimental bias, where more work had been performed within sensory regions than between regions (Scannell et al., 1995). Our structural connectome did not identify stronger connectivity within sensory regions. These inconsistencies could potentially suggest that the previously identified sensory isolation may have been a consequence experimental bias however differences in experimental method, node parcellation, connectivity model or the difference in population sample size may account for the lack of coherence between studies. Further research into the structural and functional feline connectome using various methods such as DWI MRI and resting-state functional MRI with larger sample sizes is necessary.

A limitation of our atlas was that our DTI data only had a spatial resolution of 1.5 mm^3^. The feline brain is significantly smaller than the human brain and therefore requires a higher spatial resolution data set to resolve the small structures in the brain. To account for this the DTI parameters were optimized to create the highest spatial resolution possible with our 3-Tesla system within an acceptable time frame and level of noise. Isotropic voxels of 1.5 mm^3^ were an archivable compromise at 3-Tesla with 60 diffusion directions. While the DWI population template was upsampled to 0.5 mm^3^ using advanced processing methods for structural connectome creation, these techniques use a significant amount of interpolation potentially biasing the data with artificial inflation. Higher-resolution DTI imaging has been achieved at higher field strengths or at the compromise of the number of diffusion directions (Zakszewski et al., 2014; Robinson et al., 2016; Hutchinson et al., 2017). In order to ensure that the lower resolution did not compromise the resulting tractography reconstructions, we validated our results by comparing our tract reconstructions to gross anatomic white matter dissections.

We provide a stereotaxic white matter atlas created from DTI and T1-weighted structural data. We created population maps for T1, FA, MD, RD and AD and white tract priors for the major white matter projections within the feline brain We documented the mean volume, fiber length and diffusivity parameters for each white matter prior and created a structural connectome. This atlas is available from the following online resource center: https://ecommons.cornell.edu/handle/1813/58775.2. It has potential applications of researchers using cats in neuroscientific research and for veterinarians undertaking research in clinical feline populations with spontaneous neurological disease.

## Data Availability Statement

The white matter atlas including white matter anatomic priors (Nifti and trk format), T1-weighted population template, and diffusivity maps for FA, MD, RD, and AD are available at the following online resource center: https://ecommons.cornell.edu/handle/1813/58775.2.

## Ethics Statement

The animal study was reviewed and approved by Cornell University Institutional Animal Care and Use Committee.

## Author Contributions

The following parts of the study were performed by: PJ, SC-G and AR: study design. PJ, SC-G and W-ML: data acquisition. EB: data processing. PJ and EB: tractography dissections. RP: dissection anatomic validation. PJ: manuscript preparation. PJ, RP, W-ML, AR, SC-G and EB: manuscript review.

## Conflict of Interest

The authors declare that the research was conducted in the absence of any commercial or financial relationships that could be construed as a potential conflict of interest.

## Footnotes

^1^https://fsl.fmrib.ox.ac.uk/

^2^https://www.mrtrix.org

^3^http://www.brain-connectivity-toolbox.net
