# Supplementary material for: Stereotaxic Diffusion Tensor Imaging White Matter Atlas for the in vivo Domestic Feline Brain
Source: Front Neuroanat. 2020 Feb 11;14:1. doi: 10.3389/fnana.2020.00001 (PMC7026623; doi:10.3389/fnana.2020.00001)
Supplement: Supplementary file 1 [file Table_1.pdf]

Supplementary Material

Diffusion Tensor Imaging White Matter Atlas for the Domestic Feline Brain

Johnson, Pascalau, Luh, Raj, Cerda-Gonzalez and Barry.

Frontiers in Neuroanatomy

A reference table containing connectivity metrics for each cortical region including the region number, label and lobar parcellation and their corresponding betweenness centrality, degree of connectivity, clustering coefficient and strength.

Regions	Regions Labels	Lobe Parcellation	Betweenness Centrality	Degree	Participation Coefficient	Clustering Coefficient
1	HippB	Subcortical	0	150	0.64	0.88
2	ICB	Subcortical	998	146	0.45	0.24
3	SCB	Subcortical	0	144	0.57	0.40
4	LGNB	Subcortical	0	149	0.66	0.48
5	MGBB	Subcortical	0	149	0.67	0.62
6	CNB	Subcortical	0	150	0.75	1.14
7	PAGB	Subcortical	104	133	0.37	0.29
8	CbmL	Cerebellum	7392	146	0.17	0.11
9	CbmR	Cerebellum	7482	146	0.33	0.12
10	17L	Occipital	14	150	0.47	0.42
11	17R	Occipital	0	150	0.54	0.49
12	18L	Occipital	0	150	0.57	0.64
13	18R	Occipital	0	150	0.64	0.54
14	19L	Occipital	0	150	0.61	0.66
15	19R	Occipital	0	150	0.60	0.69
16	20aL	Occipital	2626	85	0.10	0.11
17	20aR	Occipital	2362	129	0.49	0.15
18	20bL	Occipital	2498	129	0.11	0.18
19	20bR	Occipital	0	145	0.57	0.29
20	21aL	Occipital	0	139	0.53	0.17
21	21aR	Occipital	0	143	0.51	0.27
22	21bL	Occipital	266	145	0.31	0.34
23	21bR	Occipital	0	138	0.54	0.25
24	7aL	Occipital	0	144	0.63	0.45
25	7aR	Occipital	2	142	0.67	0.57
26	7mL	Occipital	1688	105	0.71	0.09
27	7mR	Occipital	406	95	0.66	0.08
28	7pL	Occipital	508	140	0.59	0.21
29	7pR	Occipital	0	142	0.50	0.48
30	ALLSL	Occipital	0	146	0.59	0.38
31	ALLSR	Occipital	0	148	0.48	0.38
32	AMLSL	Occipital	2	137	0.61	0.16
33	AMLSR	Occipital	34	135	0.36	0.23
34	PLLSL	Occipital	0	143	0.53	0.34
35	PLLSR	Occipital	0	148	0.50	0.41
36	PMLSL	Occipital	0	144	0.50	0.34
37	PMLSR	Occipital	0	144	0.62	0.31
38	DLSL	Occipital	56	123	0.37	0.15
39	DLSR	Occipital	0	139	0.52	0.31
40	VLSL	Occipital	0	131	0.40	0.16
41	VLSR	Occipital	600	98	0.58	0.08
42	CVAL	Occipital	0	150	0.56	0.44
43	CVAR	Occipital	0	147	0.54	0.58
44	SVAL	Occipital	10	134	0.69	0.15
45	SVAR	Occipital	0	144	0.64	0.29
46	PSL	Occipital	338	85	0.16	0.11

47	PSR	Occipital	1270	88	0.25	0.13
48	RSL	Subcortical	0	150	0.65	0.87
49	RSR	Subcortical	0	150	0.70	0.79
50	AEVL	Occipital	0	145	0.46	0.29
51	AEVR	Occipital	286	141	0.48	0.26
52	A1L	Temporal	634	135	0.51	0.18
53	A1R	Temporal	8	144	0.38	0.29
54	A2L	Temporal	312	147	0.52	0.48
55	A2R	Temporal	0	149	0.52	0.57
56	AAFL	Temporal	1848	140	0.52	0.29
57	AAFR	Temporal	888	145	0.60	0.44
58	PAFL	Temporal	30	137	0.30	0.23
59	PAFR	Temporal	26	146	0.39	0.37
60	vPAFL	Temporal	700	92	0.23	0.13
61	vPAFR	Temporal	500	109	0.16	0.09
62	DZL	Temporal	0	138	0.56	0.20
63	DZR	Temporal	88	133	0.35	0.37
64	INL	Temporal	0	148	0.54	0.50
65	INR	Temporal	0	149	0.46	0.50
66	TEL	Temporal	0	148	0.53	0.37
67	TER	Temporal	328	148	0.52	0.50
68	FAESL	Temporal	0	143	0.38	0.45
69	FAESR	Temporal	0	147	0.54	0.45
70	VAFL	Temporal	1478	56	0.19	0.11
71	VAFR	Temporal	2460	78	0.17	0.12
72	dPEL	Temporal	28	138	0.46	0.26
73	dPER	Temporal	0	147	0.48	0.38
74	iPEL	Temporal	40	130	0.20	0.31
75	iPER	Temporal	930	144	0.34	0.33
76	pPEL	Temporal	8	113	0.48	0.13
77	pPER	Temporal	0	142	0.51	0.37
78	vPEL	Temporal	2098	116	0.16	0.19
79	vPER	Temporal	1038	119	0.34	0.17
80	1L	Parietal	0	146	0.60	0.70
81	1R	Parietal	80	146	0.46	0.67
82	2L	Parietal	0	144	0.35	0.54
83	2R	Parietal	0	141	0.31	0.64
84	3aL	Parietal	2332	136	0.63	0.31
85	3aR	Parietal	20	141	0.59	0.44
86	3bL	Parietal	0	141	0.60	0.36
87	3bR	Parietal	0	141	0.52	0.56
88	S2L	Parietal	0	146	0.46	0.57
89	S2R	Parietal	20	142	0.34	0.85
90	S2mL	Parietal	0	144	0.56	0.37
91	S2mR	Parietal	20	145	0.65	0.38
92	S3L	Parietal	0	148	0.62	0.47
93	S3R	Parietal	30	144	0.62	0.43
94	S4L	Parietal	0	140	0.34	0.47

95	S4R	Parietal	446	139	0.48	0.35
96	S5L	Parietal	0	145	0.61	0.42
97	S5R	Parietal	0	147	0.63	0.52
98	5alL	Parietal	242	135	0.48	0.24
99	5alR	Parietal	0	138	0.48	0.37
100	5amL	Parietal	0	143	0.62	0.55
101	5amR	Parietal	0	139	0.65	0.53
102	5blL	Parietal	0	143	0.56	0.37
103	5blR	Parietal	0	139	0.60	0.39
104	5bmL	Parietal	0	143	0.66	0.38
105	5bmR	Parietal	12	142	0.63	0.56
106	5mL	Parietal	0	139	0.71	0.44
107	5mR	Parietal	6	137	0.71	0.33
108	PFdlL	Frontal	0	150	0.60	0.91
109	PFdlR	Frontal	0	147	0.47	1.14
110	PFdmL	Frontal	44	127	0.43	0.22
111	PFdmR	Frontal	742	127	0.51	0.16
112	PFvL	Frontal	0	149	0.63	0.63
113	PFvR	Frontal	226	148	0.53	0.63
114	4DeltaL	Frontal	0	138	0.56	0.30
115	4DeltaR	Frontal	1568	123	0.41	0.14
116	4fuL	Frontal	212	149	0.65	0.55
117	4fuR	Frontal	0	148	0.55	0.74
118	4GammaL	Frontal	294	125	0.60	0.16
119	4GammaR	Frontal	16	121	0.38	0.21
120	4sfuL	Frontal	512	140	0.65	0.24
121	4sfuR	Frontal	412	137	0.59	0.37
122	6aAlphaL	Frontal	0	146	0.55	0.41
123	6aAlphaR	Frontal	0	133	0.42	0.26
124	6aBetaL	Frontal	0	147	0.51	0.41
125	6aBetaR	Frontal	226	143	0.52	0.40
126	6aGammaL	Frontal	0	148	0.56	0.51
127	6aGammaR	Frontal	34	143	0.41	0.49
128	6iffuL	Frontal	3036	134	0.54	0.23
129	6iffuR	Frontal	572	122	0.40	0.23
130	PLL	Frontal	68	148	0.61	0.37
131	PLR	Frontal	116	149	0.51	0.41
132	GIL	Subcortical	124	123	0.31	0.25
133	GIR	Subcortical	224	119	0.32	0.21
134	DIL	Subcortical	40	115	0.20	0.21
135	DIR	Subcortical	272	116	0.23	0.28
136	AIdL	Subcortical	36	149	0.65	0.42
137	AIdR	Subcortical	202	148	0.52	0.53
138	AIvL	Subcortical	0	150	0.61	0.51
139	AIvR	Subcortical	0	149	0.55	0.45
140	PpL	Frontal	0	145	0.60	0.39
141	PpR	Frontal	20	148	0.47	0.41
142	CGaL	Subcortical	338	118	0.67	0.15

143	CGaR	Subcortical	344	117	0.68	0.15
144	CGpL	Subcortical	0	148	0.72	0.41
145	CGpR	Subcortical	140	146	0.65	0.40
146	36L	Subcortical	1560	125	0.20	0.26
147	36R	Subcortical	1604	141	0.27	0.30
148	MZL	Parietal	0	140	0.65	0.18
149	MZR	Parietal	0	147	0.66	0.50
150	GL	Frontal	762	131	0.39	0.28
151	GR	Frontal	0	130	0.26	0.25
